# Supplementary material for: Men's preconception health and the social determinants of health: What are we missing?
Source: Front Reprod Health. 2022 Sep 13;4:955018. doi: 10.3389/frph.2022.955018 (PMC9580700; doi:10.3389/frph.2022.955018)
Supplement: Supplementary file 2 [file Table_2.DOCX]

**eTable 2**. Examining confounding effects on the association between men’s preconception health and SDOH’s (insurance status, employment consistency, education status and race/ethnicity) using the forward selection method

**Panel 2a**. Examining confounding effects on the association between men’s preconception health and insurance status

| Regressing PhP on ***Insurance Status*** | **Sexual risk takers**  **vs**  **Lowest risk** | | **Substance users**  **vs**  **Lowest risk** | |
| --- | --- | --- | --- | --- |
|  | **OR**  **(95% CI)** | **% Change** | **OR**  **(95% CI)** | **% Change** |
| **Crude model:** Ref= yes |  |  |  |  |
| No | 1.19  (0.97, 1.46) | - | 1.50  (1.27, 1.76) | - |
| **Add: age** |  |  |  |  |
| No | 0.85  (0.70, 1.03) | 40 | 0.68  (0.58, 0.80) | 120.6 |
| **Add: age, age of sexual debut** |  |  |  |  |
| No | 0.91  (0.75, 1.11) | 30.8 | 0.74  (0.62, 0.87) | 102.7 |
| **Add: age, age of sexual debut, biological children** |  |  |  |  |
| No | 0.88  (0.72, 1.07) | 35.2 | 0.71  (0.60, 0.83) | 111.3 |
| **Add: age, age of sexual debut, biological children, union type** |  |  |  |  |
| No | 1.19  (0.98, 1.45) | 0.0 | 0.85  (0.72, 1.01) | 76.5 |
| **Add Less: age, age of sexual debut, biological children, union type, survey year**  **(Full model)** |  |  |  |  |
| No | 1.25  (1.02, 1.52) | -4.8 | 0.84  (0.71, 0.99) | 78.6 |

Notes % change= [(OR _crude_ – OR _adjusted_) / OR _adjusted_] x 100

Covariates of interest: union type, number of biological children, age of participant, age of sexual debut and survey year

**Panel 2b**. Examining confounding effects on the association between men’s preconception health and employment consistency

| Regressing PhP on ***Employment Consistency*** | **Sexual risk takers**  **vs**  **Lowest risk** | | **Substance users**  **vs**  **Lowest risk** | |
| --- | --- | --- | --- | --- |
|  | **OR** | **% Change** | **OR** | **% Change** |
| **Crude model:** Ref= Employed (stable) |  |  |  |  |
| Not employed | 1.11  (0.76, 1.61) | - | 1.19  (0.91, 1.55) | - |
| Employed (unstable) | 1.27  (1.04, 1.54) | - | 1.98  (1.71, 2.30) | - |
| **Add: age** |  |  |  |  |
| Not employed | 1.05  (0.72, 1.53) | 5.7 | 1.14  (0.86, 1.51) | 4.4 |
| Employed (unstable) | 0.99  (0.81, 1.22) | 28.3 | 1.52  (1.29, 1.78) | 30.3 |
| **Add: age, age of sexual debut** |  |  |  |  |
| Not employed | 0.97  (0.66, 1.42) | 14.4 | 1.05  (0.80, 1.39) | 13.3 |
| Employed (unstable) | 0.96  (0.79, 1.18) | 32.3 | 1.47  (1.25, 1.73) | 34.7 |
| **Add: age, age of sexual debut, biological children** |  |  |  |  |
| Not employed | 0.94  (0.64, 1.38) | 18.1 | 1.01  (0.77, 1.34) | 17.8 |
| Employed (unstable) | 0.96  (0.78, 1.17) | 32.3 | 1.46  (1.25, 1.72) | 35.6 |
| **Add: age, age of sexual debut, biological children, union type** |  |  |  |  |
| Not employed | 0.69  (0.47, 1.01) | 60.9 | 0.83  (0.63, 1.09) | 43.4 |
| Employed (unstable) | 0.74  (0.60, 0.91) | 71.6 | 1.22  (1.04, 1.45) | 62.3 |
| **Add: age, age of sexual debut, biological children, union type, survey year (Full model)** |  |  |  |  |
| Not employed | 0.68  (0.46, 1.00) | 63.2 | 0.83  (0.63, 1.10) | 43.4 |
| Employed (unstable) | 0.73  (0.59, 0.90) | 74.0 | 1.23  (1.04, 1.45) | 61.0 |

Notes % change= [(OR _crude_ – OR _adjusted_) / OR _adjusted_] x 100

Covariates of interest: union type, number of biological children, age of participant, age of sexual debut and survey year

**Panel 2c**: Examining confounding effects on the association between men’s preconception health and education status

| Regressing PhP on ***Education status*** | **Sexual risk takers**  **vs**  **Lowest risk** | | **Substance users**  **vs**  **Lowest risk** | |
| --- | --- | --- | --- | --- |
|  | **OR**  **(95% CI)** | **% Change** | **OR**  **(95% CI)** | **% Change** |
| **Crude model:** Ref= Less than high school |  |  |  |  |
| High school | 1.59  (1.15, 2.20) | - | 1.49  (1.17, 1.90) | - |
| Some college | 1.53  (1.10, 2.14) | - | 1.44  (1.12, 1.87) | - |
| College or more | 0.92  (0.67, 1.26) | - | 0.86  (0.64, 1.15) | - |
| **Add: age** |  |  |  |  |
| High school | 1.55  (1.11, 2.16) | 2.6 | 1.44  (1.12, 1.85) | 3.5 |
| Some college | 1.37  (0.98, 1.91) | 11.7 | 1.26  (0.97, 1.62) | 14.3 |
| College or more | 1.02  (0.74, 1.40) | -9.8 | 0.99  (0.74, 1.32) | -13.1 |
| **Add: age, age of sexual debut** |  |  |  |  |
| High school | 1.57  (1.12, 2.20) | 1.3 | 1.46  (1.14, 1.88) | 2.1 |
| Some college | 1.53  (1.10, 2.14) | 0.0 | 1.43  (1.11, 1.84) | 0.7 |
| College or more | 1.34  (0.96, 1.85) | -31.3 | 1.35  (1.01, 1.80) | -36.3 |
| **Add: age, age of sexual debut, biological children** |  |  |  |  |
| High school | 1.39  (0.99, 1.95) | 14.4 | 1.29  (1.00, 1.66) | 15.5 |
| Some college | 1.24  (0.88, 1.73) | 23.4 | 1.14  (0.89, 1.47) | 26.3 |
| College or more | 1.03  (0.73, 1.43) | -10.7 | 1.01  (0.76, 1.34) | -14.9 |
| **Add: age, age of sexual debut, biological children, union type** |  |  |  |  |
| High school | 1.71  (1.20, 2.43) | -7.0 | 1.48  (1.15, 1.91) | 0.7 |
| Some college | 1.63  (1.15, 2.32) | -6.1 | 1.36  (1.05, 1.77) | 5.9 |
| College or more | 1.88  (1.31, 2.71) | -51.1 | 1.47  (1.10, 1.97) | -41.5 |
| **Add: age, age of sexual debut, biological children, union type, survey year (Full model)** |  |  |  |  |
| High school | 1.74  (1.23, 2.48) | -8.6 | 1.48  (1.15, 1.90) | 0.7 |
| Some college | 1.65  (1.16, 2.36) | -7.3 | 1.36  (1.05, 1.75) | 5.9 |
| College or more | 1.94  (1.34, 2.79) | -52.6 | 1.45  (1.09, 1.94) | -40.7 |

Notes % change= [(OR _crude_ – OR _adjusted_) / OR _adjusted_] x 100

Covariates of interest: union type, number of biological children, age of participant, age of sexual debut and survey year

**Panel 2d.** Examining confounding effects on the association between men’s preconception health and Race/Ethnicity

| Regressing PhP on ***Race/Ethnicity*** | **Sexual risk takers**  **vs**  **Lowest risk** | | **Substance users**  **vs**  **Lowest risk** | |
| --- | --- | --- | --- | --- |
|  | **OR**  **(95% CI)** | **% Change** | **OR**  **(95% CI)** | **% Change** |
| **Crude model:** Ref= Non-Hispanic White |  |  |  |  |
| Non-Hispanic Black | 2.64  (2.12, 3.29) | - | 1.31  (1.07, 1.60) | - |
| Hispanic | 1.25  (0.98, 1.59) | - | 0.75  (0.62, 0.91) | - |
| Other | 0.54  (0.34, 0.86) | - | 0.61  (0.43, 0.86) | - |
| **Add: age** |  |  |  |  |
| Non-Hispanic Black | 2.60  (2.09, 3.23) | 1.5 | 1.28  (1.05, 1.56) | 2.3 |
| Hispanic | 1.22  (0.96, 1.55) | 2.5 | 0.73  (0.60, 0.88) | 2.7 |
| Other | 0.54  (0.34, 0.86) | 0.0 | 0.62  (0.44, 0.86) | -1.6 |
| **Add: age, age of sexual debut** |  |  |  |  |
| Non-Hispanic Black | 2.26  (1.79, 2.84) | 16.8 | 1.05  (0.86, 1.28) | 24.8 |
| Hispanic | 1.17  (0.91, 1.49) | 6.8 | 0.68  (0.56, 0.82) | 10.3 |
| Other | 0.60  (0.38, 0.95) | -10.0 | 0.70  (0.51, 0.96) | -12.9 |
| **Add: age, age of sexual debut, biological children** |  |  |  |  |
| Non-Hispanic Black | 2.47  (1.97, 3.10) | 6.9 | 1.14  (0.94, 1.40) | 14.9 |
| Hispanic | 1.31  (1.02, 1.67) | -4.6 | 0.75  (0.62, 0.92) | 0.0 |
| Other | 0.61  (0.38, 0.97) | -11.5 | 0.70  (0.51, 0.96) | -12.9 |
| **Add: age, age of sexual debut, biological children, union type** |  |  |  |  |
| Non-Hispanic Black | 1.96  (1.53, 2.51) | 34.7 | 0.97  (0.79, 1.18) | 35.1 |
| Hispanic | 1.20  (0.93, 1.56) | 4.2 | 0.71  (0.58, 0.87) | 5.6 |
| Other | 0.63  (0.39, 1.00) | -14.3 | 0.70  (0.51, 0.97) | -12.9 |
| **Add: age, age of sexual debut, biological children, union type, survey year (full model)** |  |  |  |  |
| Non-Hispanic Black | 1.99  (1.55, 2.54) | 32.7 | 0.96  (0.79, 1.17) | 36.5 |
| Hispanic | 1.23  (0.94, 1.59) | 1.6 | 0.71  (0.58, 0.87) | 5.6 |
| Other | 0.62  (0.39, 0.99) | -12.9 | 0.70  (0.51, 0.97) | -12.9 |

Notes % change= [(OR _crude_ – OR _adjusted_) / OR _adjusted_] x 100

Covariates of interest: union type, number of biological children, age of participant, age of sexual debut and survey year
